# Supplementary material for: Cytoplasmic FUS triggers early behavioral alterations linked to cortical neuronal hyperactivity and inhibitory synaptic defects
Source: Nat Commun. 2021 May 21;12:3028. doi: 10.1038/s41467-021-23187-9 (PMC8140148; doi:10.1038/s41467-021-23187-9)
Supplement: Supplementary file 1 — Supplementary Information [file 41467_2021_23187_MOESM1_ESM.pdf]

Scekic-Zahirovic, Sanjuan-Ruiz and collaborators

Cytoplasmic FUS triggers early behavioral alterations linked to cortical neuronal hyperactivity and inhibitory synaptic defects

Supplementary figures

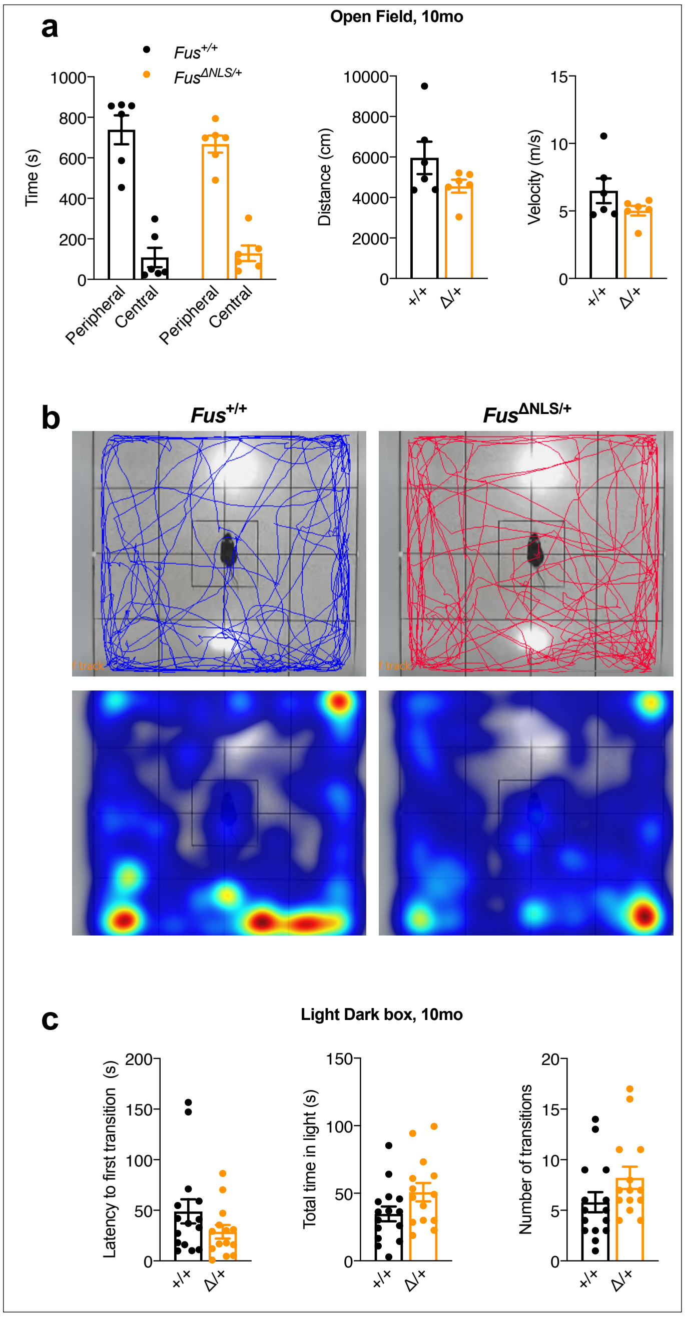

Supplementary figure 1: Supplementary behavioral characterization of *Fus*$^{\Delta NLS/+}$ mice
(**a**) Bar charts showing time spent in peripheral and central quadrants of the open field arena of 10 months old *Fus*$^{+/+}$ (black) and *Fus*$^{\Delta NLS/+}$ (orange) male mice. Total distance traveled (in centimeters) (**middle graph**) and average speed of movements (in meters per second) (**left graph**) are presented. Data are presented as mean +/- SEM. For panel a, N = 6 *Fus*$^{+/+}$ mice

and N=6 *Fus*$^{\Delta NLS/+}$ mice were analyzed. Multiple Two-sided Unpaired Student's t-tests. p = 0.84 (peripheral quadrants) and p = 0.34 (central quadrants). Unpaired t-test. p=0.13 (distance) and p = 0.16 (velocity).
(**b**) Representative images of tracking's trajectories (upper panels) and heat maps of mouse movement (lower panels) and in the open field of 10 months old *Fus*$^{+/+}$ and *Fus*$^{\Delta NLS/+}$ animals.
(**c**) Bar charts showing latency time to enter the illuminated compartment (**left graph**), total time spent in the spent in the illuminated compartment (**middle graph**), and number of transitions between dark and the illuminated compartments (**right graph**) in the dark-light box test for 10 months old *Fus*$^{+/+}$ (black) and *Fus*$^{\Delta NLS/+}$ (orange) mice. All values are represented as mean +/- SEM. For panel c, N=15 *Fus*$^{+/+}$ mice and N=14 *Fus*$^{\Delta NLS/+}$ mice were analyzed. Two-sided unpaired Student's t-test, p = 0.15 (latency) and p = 0.07 (total time) and p = 0.11 (transitions).
Source data are provided as a Source Data file.

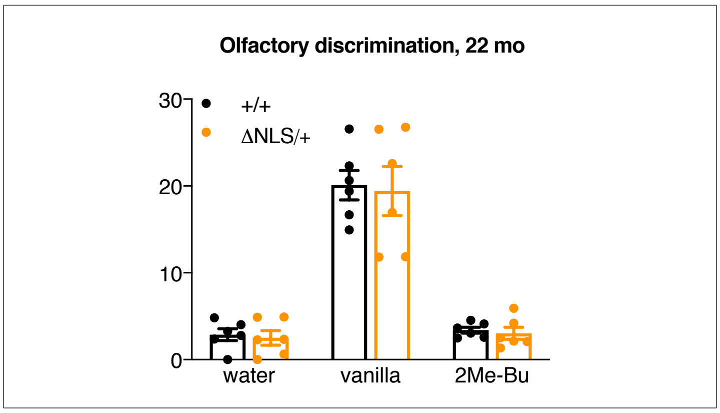

Supplementary figure 2: Preserved olfactory function in *Fus*$^{\Delta NLS/+}$ mice
Analysis of olfactory function in *Fus*$^{+/+}$ and *Fus*$^{\Delta NLS/+}$ male mice at 22 months of age. Bar graphs showing similar preference of both genotypes for "attractive" scents (vanilla) compared to water and "aversive" stimuli. N=6 *Fus*$^{+/+}$ mice and N=6 *Fus*$^{\Delta NLS/+}$ mice were analyzed. All values are presented as mean +/- SEM of time mice spent sniffing filter paper immersed in corresponding solution. Multiple two-sided unpaired Student's t-tests, p = 0.74 (water), p = 0.84 (vanilla) and p = 0.64 (2-methyl butyrate).
Source data are provided as a Source Data file.

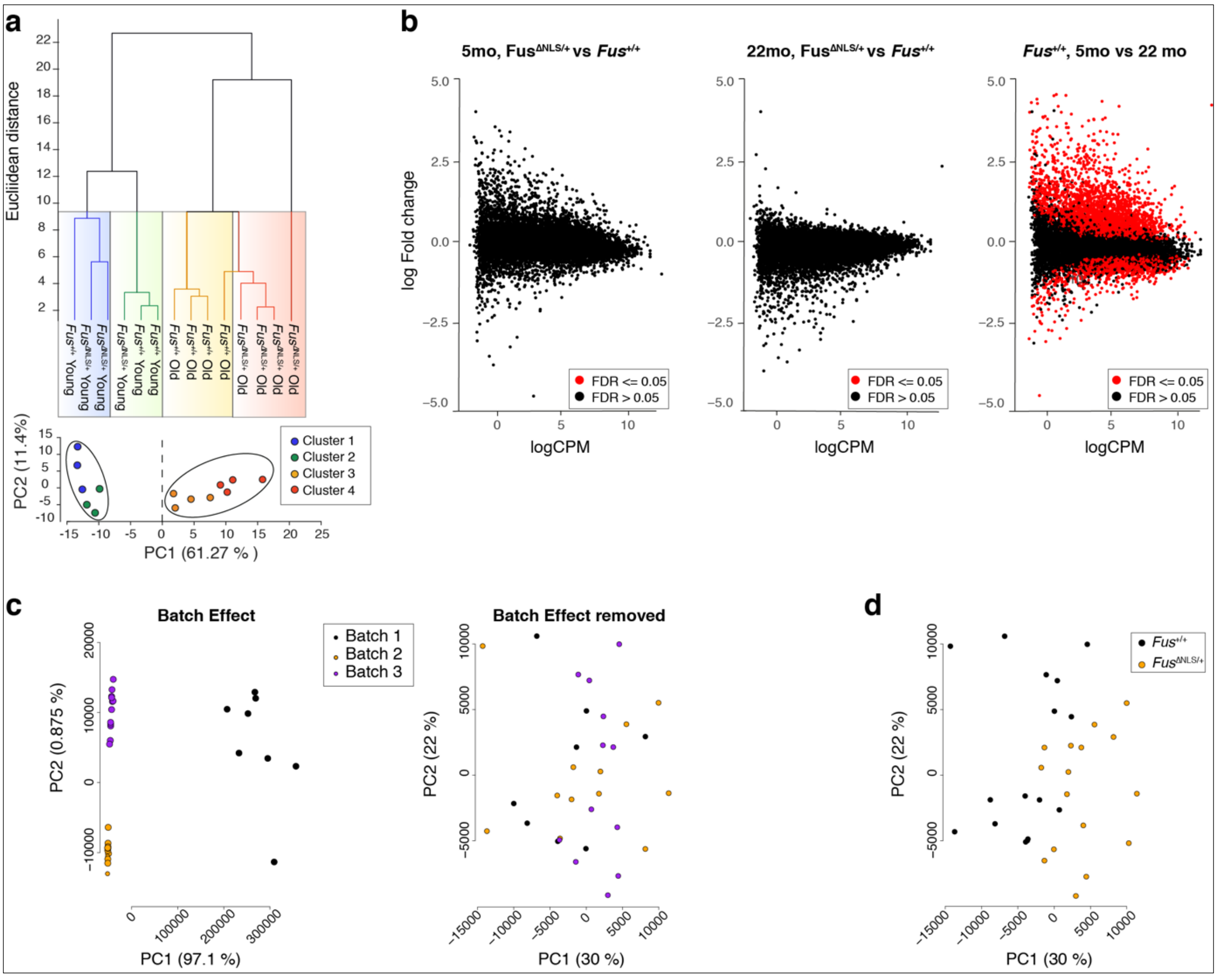

Supplementary Figure 3: Additional results of RNAseq analysis, related to Figure 5
(a) Hierarchical clustering and principal components analysis (PCA) shows that the first PCA differentiates between 5 months and 22 months old mice, while PC2 distinguishes between *Fus*$^{+/+}$ and *Fus*$^{\Delta NLS/+}$ mice only in young animals.
(b) The MA plot shows no DE genes at 5 and 22 months. However, we detect 2297 DE genes between 22 months and 5 months WT mice (FDR < 0.05).
(c-d) Negative binomial regression removes significant batch effect. After batch correction, we observe clustering of samples according to genotype on the PCA plot (**d**).

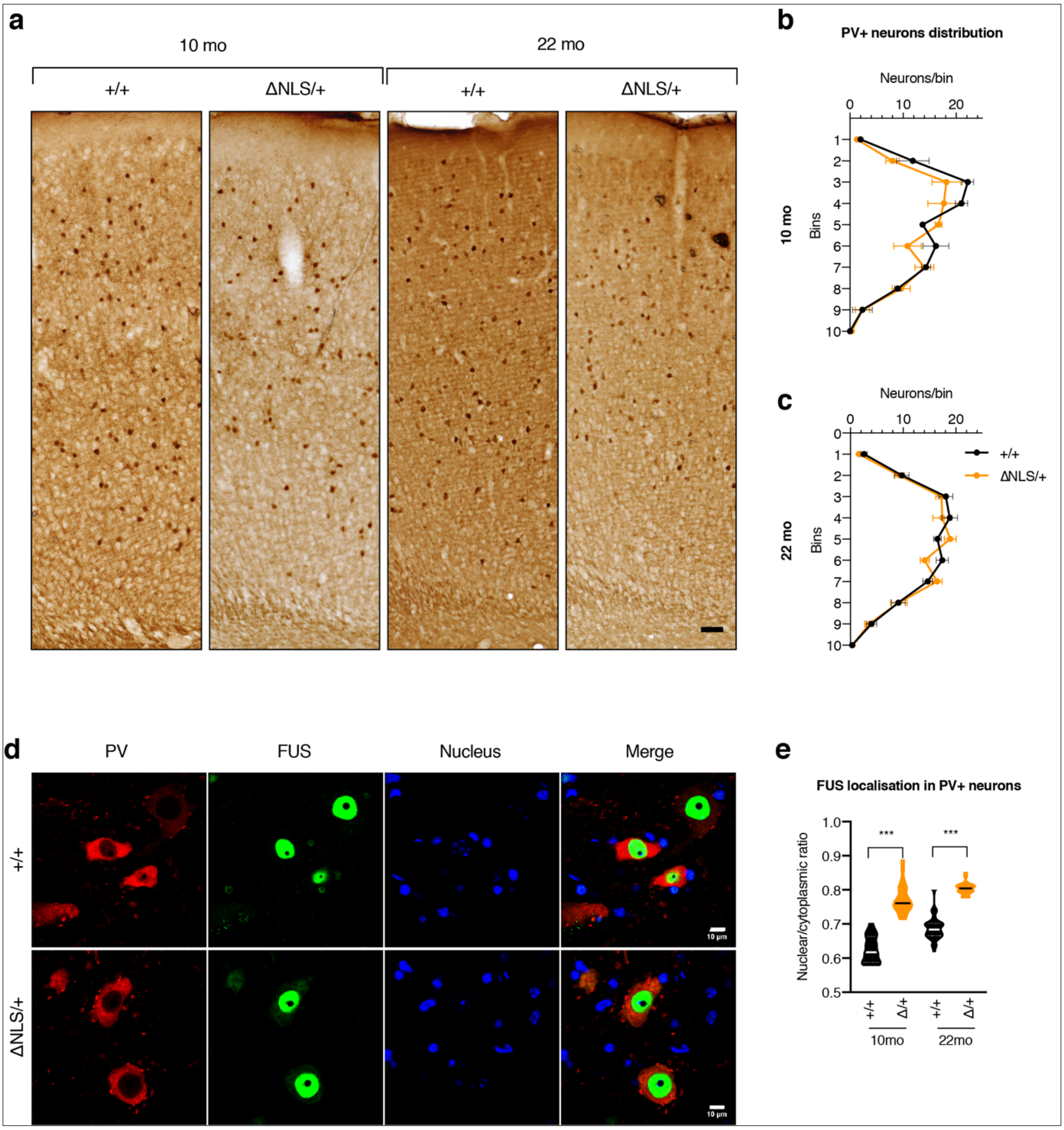

Supplementary Figure 4: Characterization of PV interneurons in *Fus*$^{\Delta NLS/+}$ mice

(**a**) Representative image of Parvalbumin immunohistochemistry of frontal cortex tissues at 10 and 22 months of age in *Fus*$^{+/+}$ (+/+) or *Fus*$^{\Delta NLS/+}$ (Δ/+) male mice. N = 3 *Fus*$^{+/+}$ mice and N = 3 *Fus*$^{\Delta NLS/+}$ mice were analyzed at 10 months of age, and N = 6 *Fus*$^{+/+}$ mice and N = 6 *Fus*$^{\Delta NLS/+}$ mice were analyzed at 22 months. Scale bar: 100μm (**b, c**) Distribution of parvalbumin+ neurons (PV+) in frontal cortex of *Fus*$^{+/+}$ (black) vs *Fus*$^{\Delta NLS/+}$ (orange) at 10 (**b**) or 22 (**c**) months of age. Data are presented as mean +/- SEM. A two-way repeated measure analysis of variance (ANOVA) (genotype * bins) was conducted to determine the effect of genotype on PV interneuron distribution. b: p=0.3618; c: p=0.8254.

(**d**) Immunofluorescence of parvalbumin (red) and FUS (green) neuronal localization in frontal cortex in *Fus*$^{+/+}$ (+/+) or *Fus*$^{\Delta NLS/+}$ (Δ/+) mice at 22 months of age. N = 6 *Fus*$^{+/+}$ mice and N = 6 *Fus*$^{\Delta NLS/+}$ mice were analyzed at 10 months of age, and N = 3 *Fus*$^{+/+}$ mice and N = 9 *Fus*$^{\Delta NLS/+}$ mice were analyzed at 22 months. Scale bar: 10μm (**e**) Violin plot displaying the distribution of the ratio between nuclear and cytoplasmic in individual PV+ neurons in the frontal cortex of

Fus$^{+/+}$ (black) or *Fus*$^{\Delta NLS/+}$ (Δ/+) mice (orange) at 10 and 22 months. N = 96 cells from 6 *Fus*$^{+/+}$ mice and N = 61 cells from 6 *Fus*$^{\Delta NLS/+}$ mice were analyzed at 10 months. N = 68 cells from 3 *Fus*$^{+/+}$ mice and N = 139 cells from 9 *Fus*$^{\Delta NLS/+}$ mice were analyzed at 22 months. Kruskal-Wallis test, followed by Dunn's for multiple comparisons. ***, P <0.0001
Source data are provided as a Source Data file.

**Age (months)**	**1**	**4-6**	**10-12**	**22**
Behavior		✓	✓	✓
In vivo imaging		✓	✓	
RNA-seq	✓	✓		✓
Synaptosomes		✓		
MRI			✓	
Histology				✓
Electron microscopy				✓

Supplementary Figure 5: Summary of experiments on a timeline.
