# Supplementary material for: Cytoplasmic FUS triggers early behavioral alterations linked to cortical neuronal hyperactivity and inhibitory synaptic defects
Source: Nat Commun. 2021 May 21;12:3028. doi: 10.1038/s41467-021-23187-9 (PMC8140148; doi:10.1038/s41467-021-23187-9)
Supplement: Supplementary file 3 — Description of Additional Supplementary Files [file 41467_2021_23187_MOESM3_ESM.pdf]

Description of Additional Supplementary Files

Supplementary Data 1: Spreadsheet including the lists of gene names for the Turquoise and the Yellow modules, related to Figure 5. Genes belonging to the modules are indicated, along with Hub genes (highlighted in Figure 5).

Supplementary Data 2: Oligonucleotide sequences for genotyping and RT-qPCRs.

Supplementary Software: Code used to perform quantification of MRI (related to Figure 4).
