# Supplementary material for: Cytoplasmic FUS triggers early behavioral alterations linked to cortical neuronal hyperactivity and inhibitory synaptic defects
Source: Nat Commun. 2021 May 21;12:3028. doi: 10.1038/s41467-021-23187-9 (PMC8140148; doi:10.1038/s41467-021-23187-9)
Supplement: Supplementary file 7 — Reporting Summary [file 41467_2021_23187_MOESM7_ESM.pdf]

nature research

Corresponding author(s): DUPUIS Luc, LIEBSCHER Sabine

Last updated by author(s): Feb 12, 2021

Reporting Summary

Nature Research wishes to improve the reproducibility of the work that we publish. This form provides structure for consistency and transparency in reporting. For further information on Nature Research policies, see our Editorial Policies and the Editorial Policy Checklist.

Statistics

For all statistical analyses, confirm that the following items are present in the figure legend, table legend, main text, or Methods section.

n/a	Confirmed	
[ ]	[x]	The exact sample size (*n*) for each experimental group/condition, given as a discrete number and unit of measurement
[ ]	[x]	A statement on whether measurements were taken from distinct samples or whether the same sample was measured repeatedly
[ ]	[x]	The statistical test(s) used AND whether they are one- or two-sided<br>*Only common tests should be described solely by name; describe more complex techniques in the Methods section.*
[x]	[ ]	A description of all covariates tested
[ ]	[x]	A description of any assumptions or corrections, such as tests of normality and adjustment for multiple comparisons
[ ]	[x]	A full description of the statistical parameters including central tendency (e.g. means) or other basic estimates (e.g. regression coefficient) AND variation (e.g. standard deviation) or associated estimates of uncertainty (e.g. confidence intervals)
[ ]	[x]	For null hypothesis testing, the test statistic (e.g. *F*, *t*, *r*) with confidence intervals, effect sizes, degrees of freedom and *P* value noted<br>*Give P values as exact values whenever suitable.*
[x]	[ ]	For Bayesian analysis, information on the choice of priors and Markov chain Monte Carlo settings
[x]	[ ]	For hierarchical and complex designs, identification of the appropriate level for tests and full reporting of outcomes
[x]	[ ]	Estimates of effect sizes (e.g. Cohen's *d*, Pearson's *r*), indicating how they were calculated

Our web collection on statistics for biologists contains articles on many of the points above.

Software and code

Policy information about availability of computer code

Data collection	in vivo two photon imaging data was acquired using SciScan (Scientifica)
Data analysis	In vivo two-photon imaging data was analyzed using custom routines written in Matlab.<br>MRI analysis was performed using custom made codes, provided as supplementary data.

For manuscripts utilizing custom algorithms or software that are central to the research but not yet described in published literature, software must be made available to editors and reviewers. We strongly encourage code deposition in a community repository (e.g. GitHub). See the Nature Research guidelines for submitting code & software for further information.

Data

Policy information about availability of data

All manuscripts must include a data availability statement. This statement should provide the following information, where applicable:

- Accession codes, unique identifiers, or web links for publicly available datasets
- A list of figures that have associated raw data
- A description of any restrictions on data availability

Source data are provided for figures 1,2, 3, 4, 6 and 7 as supplementary file.
Accession codes for RNAseq datasets (Figure 5) are provided in the manuscript (GSE166615)

Field-specific reporting

Please select the one below that is the best fit for your research. If you are not sure, read the appropriate sections before making your selection.

[x] Life sciences [ ] Behavioural & social sciences [ ] Ecological, evolutionary & environmental sciences

For a reference copy of the document with all sections, see nature.com/documents/nr-reporting-summary-flat.pdf

Life sciences study design

All studies must disclose on these points even when the disclosure is negative.

Sample size	Our sample sizes adhere to typical group sizes used in the field and was determined according to 3R recommendations of ethical committee. Statistical methods were not used to predetermine the number of animals per groups.
Data exclusions	no data was excluded for any experiment, with one exception: In resident intruder and 3 chamber tests in the 22 months old group, in which 1 wild type and 1 mutant mice were excluded as they were deviating from the rest of the cohort by more than 2 SD.
Replication	Two photon imaging: we performed the same in vivo imaging experiments in 5 and 6 mice within 2-3 field of views<br>For most behavioural experiments, we did not replicate experiments in order to adhere as closely as possible to 3R rules, and planned our experiments with sufficient experimental power to ensure the robustness of results.<br>Resident intruder test and Three chamber test were replicated in two independent groups of mice at 10 months of age as a measure undertaken to verify reproducibility of experimental findings. Experimental results were similar for both cohort analyzed and data presented in the manuscript are joint results of both cohorts.
Randomization	Mice were stratifed by genotypes and then allocated randomly to groups.
Blinding	All behavioural and analytical tests were performed blindly. Experimenters were blind to genotypes when performing behavioural tests, mice were identified by assignation of a number. Behaviour measurements and analytical analysis were performed by an experimenter blind to all genotypes in order to avoid any bias.

Reporting for specific materials, systems and methods

We require information from authors about some types of materials, experimental systems and methods used in many studies. Here, indicate whether each material, system or method listed is relevant to your study. If you are not sure if a list item applies to your research, read the appropriate section before selecting a response.

Materials & experimental systems

n/a	Involved in the study	
[ ]	[x]	Antibodies
[x]	[ ]	Eukaryotic cell lines
[x]	[ ]	Palaeontology and archaeology
[ ]	[x]	Animals and other organisms
[x]	[ ]	Human research participants
[x]	[ ]	Clinical data
[x]	[ ]	Dual use research of concern

Methods

n/a	Involved in the study	
[x]	[ ]	ChIP-seq
[x]	[ ]	Flow cytometry
[x]	[ ]	MRI-based neuroimaging

Antibodies

Antibodies used	Immunohistochemistry and Immunofluorescence - Primary Antibodies :<br>- Anti-mouse NeuN (Millipore, MAB377, 1:100, Clone A60)<br>- Anti-mouse parvalbumin (Sigma, P3088, 1:1000, Clone PARV-19)<br>- Rabbit anti-FUS (ProteinTech, 11570-1-AP, 1:100)<br>- Rabbit GABAAalpha3 (Synaptic Systems, 224 303, 1:500)<br>- Mouse Gephyrin (Synaptic Systems, 147 011, 1:500, Clone mAb7a)<br>- Guinea pig VGAT (Synaptic Systems, 131 011, 1:500, Clone 117G4)<br><br>Secondary antibodies :<br>- Biotinylated donkey anti-mouse (Jackson ImmunoResearch, 715-067-003, 1:500)<br>- Goat anti-mouse Alexa-488 (Invitrogen, A11034, 1:500)<br>- Goat anti-mouse Alexa-647 (Invitrogen, A21245, 1:500)

	Western Blotting - Primary antibodies :<br>- Anti-Synaptophysin (Abcam, ab14692, 1:1000)<br>- Anti-FUS N-ter1 (ProteinTech, 11570, 1:1000)<br>- Anti-FUS N-ter2 (Bethyl, A-300-291A, 1:2000)<br>- Anti-FUS C-ter (Bethyl, A300-294A, 1:2000)<br><br>Secondary Antibodies :<br>- Anti-Rabbit HRP (P.A.R.I.S, BI2407, 1:5000)
Validation	All primary antibodies have been validated by the companies for their use in the respective applications. We additionnally validated the 3 FUS antibodies using homozygous Fus ΔNLS and knock-out extracts in Scekic-Zahirovic et al, EMBO J, 2016.

Animals and other organisms

Policy information about studies involving animals; ARRIVE guidelines recommended for reporting animal research

Laboratory animals	Mouse line: Fus dNLS/+ and non transgenic littermates (Fus +/+) were used. Mice were bred on a C57Bl6 background. Only male mice were subjected to experimental procedures at 4, 10 and 22 months of age for behavioral study; at 9-10 months of age for using in vivo two-photon calcium imaging; and at 12 months of age for MRI.
Wild animals	study did not involve wild animals
Field-collected samples	study did not involve field collected samples
Ethics oversight	Local ethical committee from Strasbourg University (CREMEAS) approved all mouse behavioral experiments under reference number AL/27/34/02/13. The Government of upper Bavaria (license number Az 55.2-1-54-2532-11-2016) authorized in vivo two-photon imaging in anesthetized mice and „Regierungspräsidium Tübingen“ (animal license number 1431) accredited Magnetic Resonance Imaging (MRI) scans in mice.

Note that full information on the approval of the study protocol must also be provided in the manuscript.
